# Fatigue-inducing stimulation resolves myotonia in a drug-induced model

**DOI:** 10.1186/1472-6793-11-5

**Published:** 2011-02-28

**Authors:** Erik van Lunteren, Sarah E Spiegler, Michelle Moyer

**Affiliations:** 1 Pulmonary and Critical Care Medicine, Case Western Reserve University and Louis Stokes Cleveland Department of Veterans Affairs, Cleveland, OH 44106, USA; 2 Louis Stokes Cleveland Department of Veterans Affairs, Cleveland, OH 44106, USA

## Abstract

**Background:**

Slowed muscle relaxation is the contractile hallmark of myotonia congenita, a disease caused by genetic CLC-1 chloride channel deficiency, which improves with antecedent brief contractions ("warm-up phenomenon"). It is unclear to what extent the myotonia continues to dissipate during continued repetitive contractions and how this relates temporally to muscle fatigue. Diaphragm, EDL, and soleus muscles were examined in vitro during repetitive 20 Hz and 50 Hz train stimulation in a drug-induced (9-AC) rat myotonia model.

**Results:**

At the onset of stimulation, 9-AC treated diaphragm and EDL muscle had markedly prolonged half relaxation and late relaxation times (range 147 to 884 ms, 894 to 1324 ms). Half relaxation and late relaxation times reached near-normal values over the 5-10 and 10-40 subsequent contractions, respectively. In both muscles myotonia declined faster during repetitive 50 Hz than 20 Hz stimulation, and much faster than the rate of force loss during fatigue at both frequencies. Soleus muscle was resistant to the myotonic effects of 9-AC.

**Conclusions:**

In a drug-induced model of mechanical myotonia, fatigue-inducing stimulation resolves the myotonia, which furthermore appears to be independent from the development of muscle fatigue.

## Background

Slowed muscle relaxation due to persistent electrical discharges is the hallmark of myotonia [1-4]. Myotonia occurs naturally in several species (humans, goats, mice) as a result of genetic deficiency of the skeletal muscle CLC-1 channel, a disease which in humans is termed myotonia congenita [1-13]. It can also be produced experimentally by blocking muscle Cl^- ^channels of normal muscle, for example with 9-anthracene carboxylic acid (9-AC), p-chloro-phenoxy-propionic acid, or 2,4-dichlorphenoxyacetate [4,14-16]. Muscle from humans and animals with myotonia congenita has physiological, structural and biochemical alterations which transcend the loss of CLC-1 channels, including hypertrophy (goats, most humans) or atrophy (mice), alterations in fiber type composition (in particular loss of type IIB fibers, seen in most species with myotonia congenita), and altered amounts of parvalbumin [1,5,7,9,11-13,17,18]. These downstream changes may contribute to the complex abnormalities in contractile performance found in CLC-1 deficient muscle, in particular changes that occur during the contractile phase of the contraction-relaxation cycle, such as impaired force production and alterations in rate of fatigue during isometric contractions [19].

Humans with myotonia experience limb muscle stiffness which interferes particularly with the initiation of movements [3,20-22]. Involvement of respiratory muscles may lead to "breathing difficulties", dyspnea, sleep apnea, and daytime alveolar hypoventilation [23-26]. For many subjects the myotonia has an adverse effect on their ability to engage in sports [2,21-23]. The extent of myotonia, however, dissipates with repetitive contractions, the so-called warm-up phenomenon [2-4,16,21]. This has been examined in the context of a short series of low intensity contractions reducing myotonia during subsequent low intensity contractions [7,16,20,27] rather than in the context of repetitive contractions of sufficient severity to produce fatigue, such as might occur during athletic activities. The general purpose of the present study was therefore to examine myotonia during fatigue-inducing stimulation. Based on previous studies examining the decline of myotonia in response to non-fatiguing contractions, the specific hypotheses of the present study were that a) myotonia declines more rapidly than force during fatigue-inducing stimulation, and b) the rate of decline of the myotonia during fatigue-inducing stimulation is affected by the frequency of stimulation producing fatigue (specifically higher stimulation frequencies augment the rate of dissipation). In order to avoid the effects of downstream structural and biochemical changes (eg., alterations in fiber type composition and atrophy or hypertrophy [1,5,7,9,11-13,17,18]) resulting from genetic CLC-1 channel deficiency that can affect muscle fatigue properties in manners apart from the myotonia itself, the present study utilized Cl^- ^channel blockade with 9-AC to produce myotonia in otherwise normal muscle rather than testing muscle that is genetically deficient in Cl^- ^channels. Muscles pertinent to breathing (diaphragm), fast twitch movements (EDL), and slow twitch movements (soleus) were tested to determine whether 9-AC has varying effects on muscles with different fiber type compositions.

## Methods

Studies were performed using adult male Sprague-Dawley rats (n = 35, weight = 390 ± 15 grams). All protocols were approved by the Institutional Animal Care and Use Committee and conformed to animal care guidelines established by the National Institutes of Health. Rats were well-anesthetized using a rodent anesthetic cocktail (initial dose, ketamine 21-30 mg/kg, xylazine 4.3-6.0 mg/kg and acepromazine 0.7-1.0 mg/kg, with supplemental smaller doses given as needed to produce and maintain a deep level of anesthesia). The EDL and soleus muscles were removed from the legs with intact tendons, and the diaphragm muscles were removed with intact rib origins and central tendon insertions. Contractile studies were performed in physiological solution consisting of (in mM): 135 NaCl, 5 KCl, 2.5 CaCl2, 1 MgSO4, 1 NaH2PO4, 15 NaHCO3, and 11 glucose, with the pH adjusted to 7.35-7.45 at 37°C while being aerated with 95% O_2_-5%CO_2_. The diaphragm muscle was cut into small strips parallel to the fiber direction keeping origin and insertion intact (strip width ~5 mm), and mounted vertically in a double-jacketed chamber while the temperature was maintained at 37°C. The soleus and EDL muscles were similarly mounted in the same chamber by the attached tendons, except that they were left whole and not cut into strips like the diaphragm. A pair of platinum electrodes was placed parallel to the diaphragm muscle strips or limb muscles, and supramaximal voltages were delivered with a pulse width of 1 ms. The lengths of the muscle samples were adjusted until the twitch force was maximized (optimal length), and kept at this length for the duration of the contractile study.

Ten minutes after the diaphragm muscle or limb muscle (at optimal length) had equilibrated to the Kreb's solution, three twitch contractions were recorded 10 seconds apart to determine the baseline force of the muscle. Three minutes thereafter, another three twitches were recorded 10 seconds apart to verify stability of the muscle sample (defined as <5% variability in force between first and second set of twitches). Subsequently 1 ml Kreb's solution containing 9-AC was added to half of the muscle samples to obtain a bath concentration of 100 μM, and to the other half of the muscle samples 1 ml of the Kreb's solution without 9-AC was added. Twenty minutes later repetitive train stimulation was initiated. This consisted of intermittent 20-Hz or 50-Hz train stimulations, with a train length of 0.33 s and one train every three seconds. The relatively long inter-train interval was needed due to the markedly slowed rate of relaxation in the presence of 9-AC, as this allowed time for muscle force to return back to baseline prior to the subsequent train. Due to the slow isometric kinetics of the soleus muscle, contractions have a high degree of fusion during 50 Hz stimulation, impairing the ability to measure contraction and relaxation times. Therefore soleus was only studied during 20 Hz stimulation.

**Figure 1:**
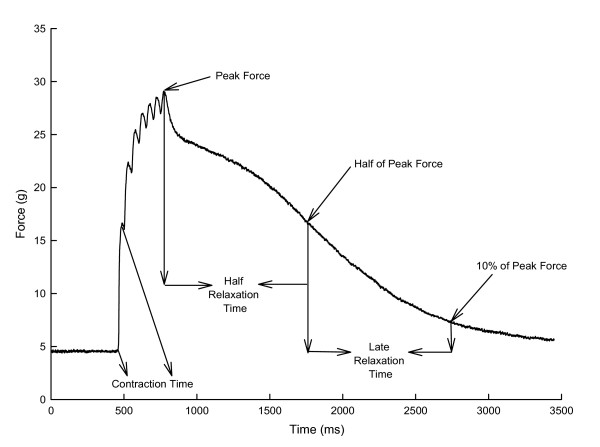
**Parameters measured for a diaphragm myotonic contraction at 20 Hz**. The baseline is passive force related to stretching the muscle to optimal length, and all other active force values were quantified relative to baseline.

Muscle contractile performance was assessed with respect to four parameters (Figure 1), and in all instances this was relative to baseline passive tension. Peak force was defined as the largest value that occurred during the portion of the contraction while the muscle was being electrically stimulated (and thus did not include mechanical myotonia even if mechanical myotonia exceeded force during active electrical stimulation). These force values were normalized relative to twitch force before drug addition to factor out the effects of variability among muscle samples in absolute force related to variability in animal size and muscle sample size, similar to previous studies of a similar nature [11,15,19,22]. Contraction time was defined as the time from the onset of force production to the top of the first peak of the non-fused contraction; values for contraction time are not presented in those instances in which a distinct first peak was not discernable (which was the case for 50 Hz stimulation for the diaphragm). Half-relaxation time was the time required for force to decrease from the peak value at the end of electrical stimulation to 50% of this value. Late relaxation time was the time for force to decline from 50% of peak to 10% of peak. These four values were quantified for a total duration of six minutes. All data presented are means and SD. Statistical analysis of paired data was performed with a paired t-test. Statistical analysis of data obtained during repetitive stimulation was performed with analysis of variance for repeated measures followed by the Newman-Keuls post-hoc test in the event of significance by analysis of variance. The level for statistical significance was set at P < 0.05 (two tailed).

## Results

### Diaphragm

The effects of 9-AC on diaphragm force, contraction time, half relaxation time and late relaxation time at the outset of repetitive stimulation are listed in Table 1. 9-AC increased force and contraction time during 20 Hz but not 50 Hz stimulation. Half relaxation and late relaxation times increased during both 20 Hz and 50 Hz stimulation, reaching values in the hundreds of milliseconds. Furthermore, values were generally longer for late relaxation (~1 to 1.5 seconds) than for the half-relaxation time (~0.5 to 1 second). None of these parameters changed at the outset of repetitive stimulation in the control muscle samples which were not treated with 9-AC.

**Table 1:** The outset of repetitive stimulation during 20 Hz and 50 Hz stimulation.

Stimulation Measurement	Frequency Stimulation	Before Krebs	After Krebs	Before 9-AC	After 9-AC
**Peak force (% of initial)**	**20 Hz**	138 ± 8	142 ± 10	155 ± 21	315 ± 53*

	**50 Hz**	319 ± 65	345 ± 91	293 ± 47	356 ± 58

**Contraction time (ms)**	**20 Hz**	23.7 ± 2.3	23.8 ± 1.2	23.2 ± 2.8	44.8 ± 6.7*

	**50 Hz**	20.1 ± 0.9	20.6 ± 1.3	20.0 ± 0.7	20.7 ± 1.2

**Half relaxation time (ms)**	**20 Hz**	26.0 ± 4.4	26.5 ± 1.7	29.2 ± 5.0	883.5 ± 296.0*

	**50 Hz**	26.4 ± 1.6	27.9 ± 2.6	28.6 ± 3.2	421.7 ± 308.3*

**Late relaxation time (ms)**	**20 Hz**	45.5 ± 11.8	51.4 ± 10.4	73.6 ± 50.8	1324.4 ± 339.2*

	**50 Hz**	32.0 ± 6.0	34.5 ± 3.9	28.6 ± 8.0	1142.6 ± 504.9*

The effect of Kreb's solution and 9-AC on diaphragm muscle at the outset of repetitive stimulation on peak force, contraction time, half relaxation time, and late relaxation time during 20 Hz and 50 Hz stimulation. Values are means ± SD. Asterisks indicate significant differences between before and after 9-AC data.

**Figure 2:**
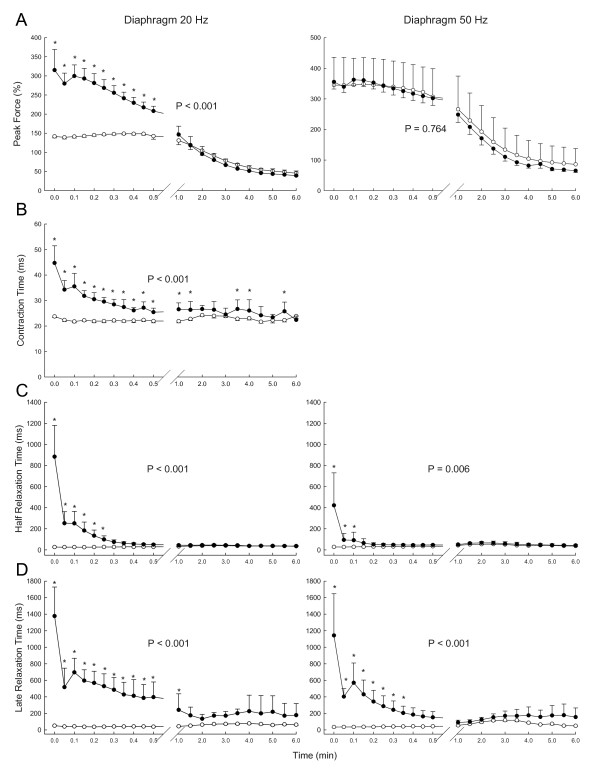
**The effect of 9-AC on force production, contraction time, half relaxation time and late relaxation time in diaphragm muscle during repetitive 20 Hz and 50 Hz train stimulation**. Contraction time was not measurable at 50 Hz. Values are means and bars indicate SD. P values are the results of analysis of variance; asterisks indicate significant differences found by the Newman-Keuls test in the event of significance by analysis of variance.

Force values for diaphragm during repetitive 20 and 50 Hz stimulation are depicted in Figure 2A. During 20 Hz repetitive stimulation, force declined rapidly in muscles that were treated with 9-AC after the initial 9-AC-induced augmentation. At one minute into the fatigue-inducing stimulation and all points thereafter, force values were equivalent in 9-AC treated and untreated muscles. In contrast, force values did not differ for drug-treated and untreated diaphragm at any time during 50 Hz stimulation. Contraction time also was prolonged by 9-AC during 20 Hz stimulation (Figure 2B) and not measurable during 50 Hz stimulation because successive stimulation was 20 ms apart and contraction time was greater than 20 ms. This prolongation waned with repetitive fatigue-inducing stimulation, but did not fully dissipate over the course of six minutes.

Both the half relaxation time (Figure 2C) and the late relaxation time (Figure 2D) were prolonged substantially by 9-AC, but eventually declined to normal or near-normal values during fatigue-inducing stimulation. The decline was more precipitous for the early compared to the late phase of relaxation as well as during 50 Hz compared with 20 Hz stimulation. The rate at which myotonia resolved was much faster than the rate at which force decreased, in that myotonia was almost completely resolved over the first 60 seconds of stimulation whereas force continued to decline for the full duration of stimulation (compare Figures 2C and 2D with Figure 2A). Furthermore, during 50 Hz stimulation the mechanical myotonia was substantially resolved before there was any appreciable force loss.

### Limb Muscles

**Figure 3:**
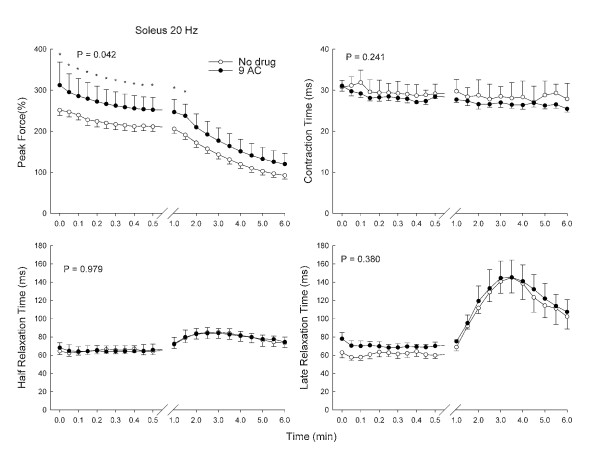
**The effect of 9-AC on peak force, contraction time, half relaxation time, and late relaxation time in the soleus muscle during repetitive 20 Hz train stimulation**. Values are means and bars indicate SD. P values are the results of analysis of variance; asterisks indicate significant differences found by the Newman-Keuls test in the event of significance by analysis of variance.

Soleus muscle did not demonstrate myotonia in response to 9-AC (data shown for 20 Hz stimulation in Figure 3). There was a mild increase in force (Figure 3A), but contraction time (Figure 3B), half relaxation time (Figure 3C) and late relaxation time (Figure 3D) were unaffected.

**Figure 4:**
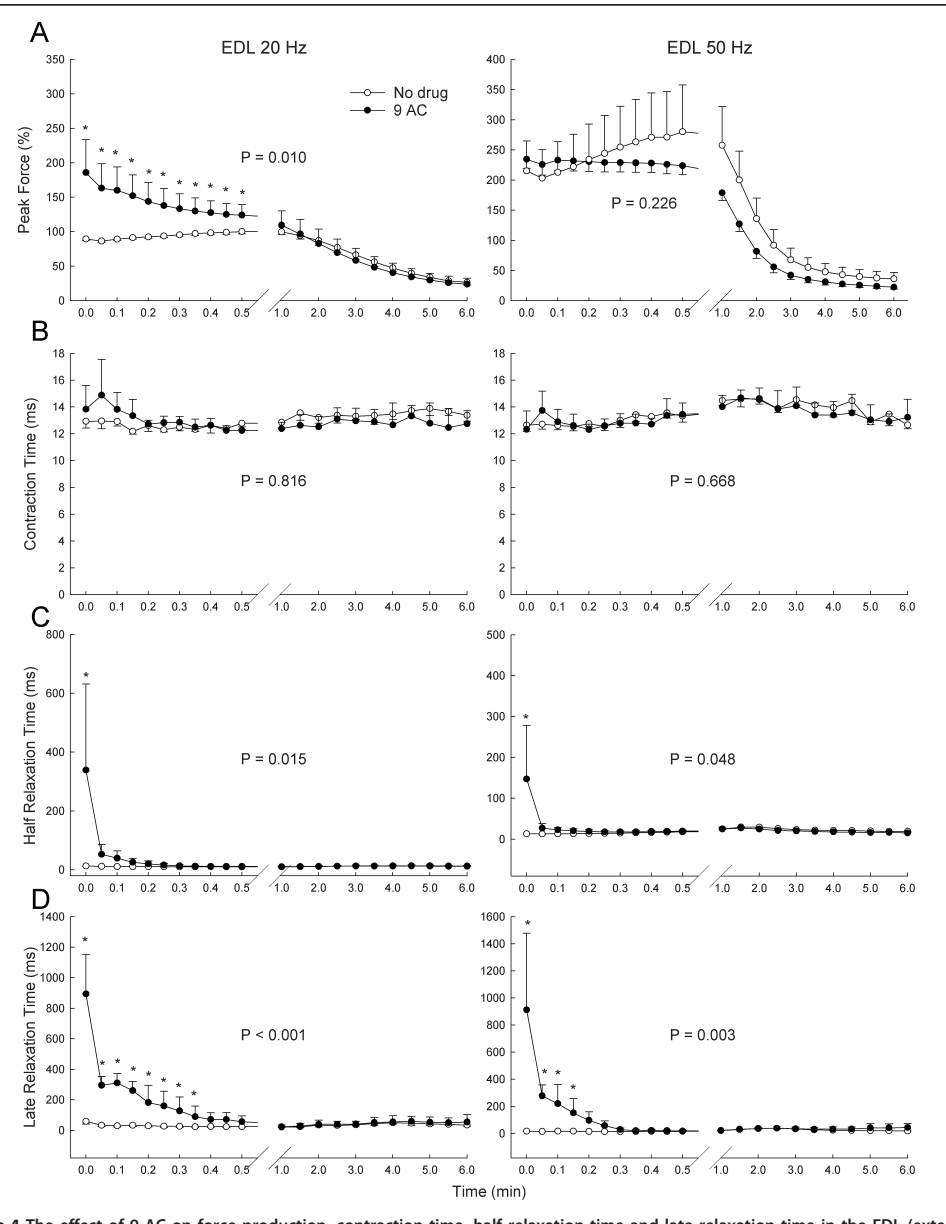
**The effect of 9-AC on force production, contraction time, half relaxation time and late relaxation time in the EDL (extensor digitorum longus) muscle during repetitive 20 Hz and 50 Hz train stimulation**. Values are means and bars indicate SD. P values are the results of analysis of variance; asterisks indicate significant differences found by the Newman-Keuls test in the event of significance by analysis of variance.

In contrast, the response of EDL was similar in most respects to that of the diaphragm. Peak EDL force increased during 20 Hz but only minimally so during 50 Hz stimulation (Figure 4A), and for 20 Hz stimulation this waned over time with repetitive stimulation. However, contraction time was not altered by 9-AC at either stimulation frequency (Figure 4B). Both half-relaxation time (Figure 4C) and late relaxation time (Figure 4D) of the EDL were prolonged by 9-AC. All relaxation times prolonged by 9-AC normalized rapidly during repetitive stimulation, and this occurred faster than the rate at which force declined, in that myotonia was almost completely resolved over the first 30 seconds of stimulation whereas force continued to decline for the full duration of stimulation (compare Figures 4C and 4D with Figure 4A). As seen with the diaphragm, during 50 Hz stimulation the mechanical myotonia was substantially resolved before there was any appreciable force loss.

## Discussion

The inability of muscles to relax normally after voluntary contractions is characteristic of the disease myotonia. The results of this study showed that mechanical myotonia decreases rapidly during fatigue-inducing stimulation. Furthermore, myotonia was found to decrease faster than force in both the diaphragm and EDL muscles. The normalization of relaxation times was slightly faster at a higher frequency (50 Hz) than a lower frequency (20 Hz) in the diaphragm muscle; however this may have been due to the effects of myotonia being less during 50 Hz than 20 Hz stimulation. Furthermore, the resolution of myotonia was also slightly faster in the EDL muscle than the diaphragm muscle, although again possibly because of differences in the magnitude of the myotonia. Both the diaphragm and EDL muscles had much larger degrees of myotonia than the soleus in response to 9-AC. In addition, force production increased at the lower stimulation frequency (20 Hz) in the diaphragm and EDL, and increased slightly in the soleus at this frequency, but did not increase at the higher frequency (50 Hz) in any of the three muscles with 9-AC. Contraction time increased only in the diaphragm during 20 Hz stimulation.

Myotonia, whether genetically- or drug-induced, decreases during repeated contractions, this resolution being termed the "warm up" phenomenon. This has been described in clinical reports of humans with myotonia congenita and is also apparent when observing the behavior of genetically myotonic mice [2,3,21]. Physiological manifestations when tested experimentally include improved rate of muscle relaxation and diminution of the duration and intensity of persistent electromyographic activity following repetitive muscle activation.

One of the early experimental studies of the warm up phenomenon was that performed by Senges and Rudel [16] in a model of myotonia induced by 2,4-dichlorphenoxyacetate. They examined the effects of a conditioning tetanus preceding a test contraction by 0.5, 1, 2 and 4 seconds. The shortest time interval virtually abolished myotonia, with the effect diminishing markedly as the time interval increased.

The warm up phenomenon was described by Heller et al.[7] in the original report of genetically myotonic mice. This was demonstrated based on electromyographic recordings from affected muscle in response to direct peripheral nerve stimulation rather than on measurements of muscle force. Heller and colleagues [7] described the myotonia as declining with repeated trials at 10 second intervals, but recovering when the muscle has rested for one minute.

The warm-up phenomenon was also studied by Heimann et al [27]. EDL muscles from myotonic ADR mutants and ADR-MDX double mutants were shown to have similar degrees of myotonia as quantified by the myotonia index [28] (myotonia index of 0.49 vs. 0.57, respectively). Furthermore, they had similar degrees to which the myotonia improved when contractions were preceded by a series of single twitches and incomplete tetanic stimulations. However, the study also found that myotonia symptoms were more pronounced in ADR than ADR-MDX muscle, but ADR-MDX mice had higher levels of weight reduction and premature death. In contrast, for the soleus the ADR mutant had a significantly higher myotonia index than the ADR-MDX mutant (myotonia index of 0.90 vs. 0.61). Data for the warm up phenomenon were not depicted for the soleus.

Van Beekvelt et al. [21] studied three humans with myotonia and defined the warming-up phenomenon as the force recovery phase after initial paresis during a sustained voluntary contraction. This study hypothesized that warming-up was due to the enhanced activation of Na+-K+-ATPase during exercise, and used ouabain (a Na+-K+-ATPase inhibitor) to test this. However, it was found that ouabain infusion did not prevent recovery from transient paresis. Therefore, it was concluded that the warm-up phenomenon was not due to Na+-K+-ATPase.

Taken together, the above studies provide a comprehensive picture of the manner in which myotonia improves following brief contractions, but these studies had not examined changes in myotonia during the course of repetitive fatigue-inducing contractions. The latter issue has been examined to a limited extent in a previous study from our lab in which we examined isotonic contractions of diaphragm muscle from genetically myotonic mice [19]. Diaphragm from these mice had lesser degrees of myotonia than seen in the diaphragm of the present study. Total relaxation time during a singe train contraction ranged from ~0.2 to 0.6 seconds in contrast to values exceeding 2 seconds with 9-AC in the present study. In the genetically myotonic mice, repetitive train stimulation at a frequency of 50 Hz resulted in a rapid reduction in relaxation time over the course of 30 seconds, with minimal changes thereafter during the subsequent 30 seconds.

The present study expands on our previous findings [19] in several manners. We found that the reduction of myotonia during fatigue-inducing contractions occurs during isometric as well as isotonic contractions, and in the drug-induced myotonia model as well as genetic myotonia. Furthermore, the present study indicates that the resolution of myotonia varies as a function of the frequency of muscle stimulation. In addition the extent of drug-induced myotonia differed among the three muscles studied, with the slow fiber type predominance of the soleus appearing to have had a protective effect. Finally, the present data indicate that the time course of the warm-up phenomenon is much faster than that of force loss during fatigue-inducing contractions.

One of the limitations of the present study is that the studies were done in vitro, under conditions in which there is no blood flow and thus no delivery of nutrients and/or impaired removal of potentially adverse metabolites. Thus the development of fatigue may have occurred faster in the drug-induced myotonia model than would occur in vivo in humans or animals with genetic CLC-1 chloride channel deficient myotonia. A second limitation is that the genetic muscle diseases that cause myotonia are a heterogeneous group which include not only two variants of CLC-1 deficient myotonia but also myotonic dystrophy. Several of these disorders have features in addition to the myotonia, which for some includes muscle weakness. Among these additional features, muscle weakness in particular may impact the rate at which fatigue develops, thereby changing the temporal relationship between the improvement of the myotonia and the reduction of force in response to repetitive vigorous contractions.

## Conclusions

Fatigue-inducing contractions were shown to dissipate the amount of mechanical myotonia induced by the chloride channel blocker 9-AC. However the rate at which this occurred was considerably faster than the drop in force over time, consistent with different mechanisms accounting for the warm up phenomenon and the production of fatigue. From a clinical perspective this suggests that subjects with myotonia need not warm up to a sufficiently large extent to produce muscle fatigue in order for the myotonia to dissipate, but nonetheless suggest a role for vigorous rather than more modest contractions as a means for resolving the muscle myotonia more quickly.

## Abbreviations

9-AC: 9-anthracene carboxylic acid.

## Authors' contributions

EvL conceived of, and designed the study, participated in statistical analysis, and helped to draft the manuscript. SS participated in the design of the study, collection of data, statistical analysis, and helped to draft the manuscript. MM participated in the design of the study, collection of data, and statistical analysis. All authors read and approved the final manuscript.
